# The prevalence of diabetes among tuberculosis patients in Denmark

**DOI:** 10.1186/s12879-022-07048-4

**Published:** 2022-01-19

**Authors:** Franziska Grundtvig Huber, Kristina Langholz Kristensen, Inge Kristine Holden, Peter Henrik Andersen, Banoo Bakir, Anja Jørgensen, Hans Johan Niklas Lorentsson, Karen Bjorn-Mortensen, Isik Somuncu Johansen, Pernille Ravn

**Affiliations:** 1 Zealand University Hospital, Køge, Denmark; 2 Gentofte Hospital, Copenhagen, Denmark; 3 Rigshospitalet, Copenhagen, Denmark; 4 Odense University Hospital, Odense, Denmark; 5 Statens Serum Institut, Copenhagen, Denmark; 6 Steno Diabetes Center Copenhagen, Copenhagen, Denmark; 7 Greenlands Center for Health Research, Ilisimatusarfik, Nuuk, Greenland; 8 Mycobacteria Centre for Research Southern Denmark, Odense, Denmark; 9 Department of Emergency Medicine, Nykøbing F. Hospital, Nykøbing Falster, Denmark

**Keywords:** Tuberculosis, Diabetes, Greenland, Denmark, Risk factors

## Abstract

**Setting:**

It is estimated that 25% of the world’s population are infected with *Mycobacterium tuberculosis* and that 463 million people are living with diabetes mellitus (DM), a number that is increasing. Patients with DM have three times the risk of developing tuberculosis (TB) and there is significant interaction between DM and TB, suggesting that DM affects not only risk of TB but also TB presentation, treatment response and outcome.

**Objective:**

The aim was determining the prevalence of DM among TB patients in Denmark and to assess risk factors.

**Design:**

Patient files from all notified TB cases in Denmark from 2009 to 2014 were retrospectively assessed.

**Results:**

In total, 1912 patients were included and 5.0% had DM. Patients with DM were older, had more comorbidities, came from outside Denmark, and had a higher mortality compared to non-DM-patients. None of the patients from Greenland had DM. Patients with low socio-economic status had a low prevalence of DM. We found a higher prevalence of DM among Danish-born < 54 year and migrant ≥ 75 year compared to a Danish background population.

**Conclusion:**

We found a higher prevalence of DM among TB patients with known risk factors, and a surprisingly low prevalence among patients with low socioeconomic status and patients from Greenland.

## Introduction

Tuberculosis (TB) is one of the deadliest infectious diseases worldwide, with 25% of the population worldwide infected by *Mycobacterium tuberculosis*. It is estimated that 10 million people develop TB each year and over 1.5 million die from TB [1, 2]. Since 2000, TB mortality has decreased by 27% [1]. Diabetes mellitus (DM) incidence is rising worldwide. In 2019 it was estimated that 463 million people were living with DM and projections of the rise in DM prevalence are alarming [3].

The association between TB and DM is well established. Already in 1947 one study described that almost 50% of patients with DM developed pulmonary TB and patients with poor diabetic control were more likely to develop active TB [4]. More recent studies estimate that approximately 15% of new TB cases could be attributed to DM [5–8]. One systematic review found that patients with DM had three times increased risk of TB regardless of background TB incidence, study region, and study design [9]. Patients with DM and TB are more likely to have severe symptoms, higher mortality, higher risk of relapse and longer sputum conversion time to normal compared with patients without DM [10–13]. DM is described by the World Health Organisation as a neglected, important and re-emerging risk factor for TB [1, 14]. It is expected that almost 80% of global adult DM cases occur in developing countries and the convergence of these two epidemics may lead to an increase in incidence of TB [15]. In Denmark, most patients with TB come from regions with high prevalence of DM, but data on the burden of DM among TB patients in Denmark is limited*.* To our knowledge, only one study has been conducted among a Danish population addressing the prevalence of TB among DM patients. The present study, in contrast, aims to determine the prevalence and risk factors of DM among patients with TB in Denmark.

## Methods

### Study population and study area

The study was carried out as a nationwide retrospective register-based cohort study including all adult patients (> 18 years) notified with TB in Denmark from January 1st 2009 to December 31st 2014. The population has been previously described in detail by Holden et al. [16]. In brief, all medical records were reviewed for socio-demographics, clinical characteristics, diagnosis, treatment of TB and outcome. Patients were diagnosed with TB by a positive microscopy, polymerase chain reaction (PCR) or sputum culture. Both patients with pulmonary TB (PTB) and extrapulmonary TB (ETB) were included in the study. We did not have access to individual information on drug-resistance. Patients under the age of 18 years were excluded from the study. If a patient did not have a valid CRP number which gave us access to information on comorbidities through National patients register (LPR) they were excluded. Some patients were registered with more than one TB diagnosis, only the first reported TB incident was included in the study.

### Ethical approval

The study was carried out in accordance with the STROBE guidelines and regulations and approved by the Danish Patient Safety Authority (3-3013-2531/1) and the Danish Data Protection Agency (VD-2018-180 and 18/40838). In accordance with Danish law, observational studies performed in Denmark do not need approval from the Danish Ethics Committee or written consent from patients (Danish Scientific Ethical Committee Act §14). All analyses are presented anonymously.

### Data collection

In Denmark, TB is monitored by the Department of Infectious Epidemiology and Prevention at Statens Serum Institut (SSI) and TB cases were retrieved from there. All culture-based analysis is performed here. We chose the first time of diagnosis for patients registered with multiple TB registration dates.

Information on baseline characteristics, risk factors, and mortality were obtained from patient records. Information on region of origin was retrieved from the Department of Infectious Disease Epidemiology and Prevention, SSI and information on comorbidities was obtained from the National Patients Register in form of a Charlson Comorbidity Score [17] based on patients discharge diagnoses. Migrant status was defined as patients born abroad or those born in Denmark for whom one or both parents had been born abroad, including patients or parents born in Greenland.

Sociodemographic information was collected from medical records, and excessive use of alcohol was quantified according to the Danish Health Authorities recommendations. A patient was registered as having DM if they had an ICD10 diagnose code of DM or if it was mentioned in the patient record that the patient had diabetes. In Denmark a patient is diagnosed with DM if they have a random blood sugar measurement of > 11.1 mmol/L, a fasting blood sugar measurement > 7 mmol/L or if a patient has a HbA1c measurement of > 48 mmol/mol, or an impaired oral glucose tolerance test.

### Statistics

Data were described as count (%) for categorical variables and mean [standard deviation (sd)] or median [interquartile range (IQR)] for continuous variables. χ^2^ and t-test were used to describe differences between different groups of patients as appropriate. Risk factors for known DM were assessed using logistic regression and multiple logistic regression (causal-based approach). A p-value < 0.05 was considered statistically significant. Data were analysed using R Studio (Version 1.1.456 and version 1.2.5042).

## Results

**Fig. 1:**
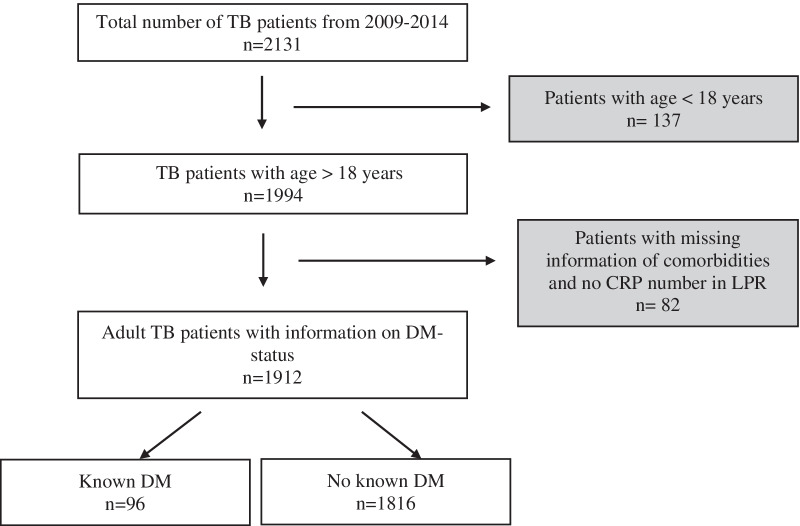
Inclusion of patients in the study, showing the number of patients in each category and number of patients excluded

In total, 2131 patients were registered with TB, 137 were < 18 years old and 82 had incomplete data or could not be found in the registers and were excluded (Fig. 1). Thus, 1912 patients with TB were included in the analysis, 668 (34.9%) were born in Denmark, 452 (23.6%) in Asia, 346 (18.1%) in Greenland, 249 (13.0%) in Africa, 195 (10.2%) in Europe outside Denmark and 11 (0.57%) came from either North- or South America (Table 1). We found an overall DM prevalence of 5.0% (n = 96). The prevalence of DM remained relatively stable through the study period, ranging from 4.3% in 2009 to 5.0% in 2014.

**Table 1:** Percentage of TB patients with known DM according to countries or regions of origin

	Total number of patients with TB	Number of patients with TB and DM	Percentage of patients with TB and DM (%)
Denmark	668	41	6.14
Europe—outside Denmark	195	15	7.7
Africa	249	16	6.4
Asia	452	24	5.3
Greenland	346	0	0.0
North America	5	0	0.0
South America	6	0	0.0
Total	1912	96	

The DM prevalence among Danish-born TB patients was 6.1%, among TB patients from the rest of Europe 7.7%, and in patients from Asia and Africa 5.3% and 6.4%, respectively. No TB patients from Greenland, South America, or North America had a DM diagnosis (Table 1).

### Characteristics for TB patients stratified by DM status

TB patients with DM were significantly older than TB patients without DM, had a higher Charlson Comorbidity Score and had a much higher mortality (17.7% versus 6.8%) (p < 0.01) (Tables 2 and 3). In contrast, patients with DM were less likely to be homeless, have excess use of alcohol, smoke cigarettes or use cannabis (p < 0.01).

**Table 2:** Baseline characteristics for TB-patients stratified by DM status

Patients	Known DM (n = 96)		No known DM (n = 1816)		p-value
	n	%	n	%	
Age, median (IQR)	57.0 (47;70)		44.0 (32; 54)		< 0.01
Sex					
Male	65	67.7	1125	61.9	0.31
Socio-economic factors					
Homeless	4	4.2	194	10.7	< 0.01
Prison	0	0	34	1.9	0.24
Alcohol use	19	19.8	671	36.9	< 0.01
Narcotics use	7	7.3	155	8.5	0.81
Cannabis use	5	5.2	357	19.7	< 0.01
Tobacco use	41	42.7	1074	59.1	< 0.01
Country or region of origin					
Denmark	41	42.7	627	34.5	0.13
Africa	16	16.7	224	12.3	0.28
Europe	15	15.6	180	9.9	0.10
Asia	24	25.0	428	23.6	0.84
Greenland	0	0.0	346	19.1	< 0.01
North or South America	0 0	0.0 0.0	5 6	0.3 0.3	1 1
Type of TB					
Pulmonary TB	74	77.1	1420	78.2	0.90
Extrapulmonary TB	22	22.9	396	21.8	0.90
TB > 1 site	10	10.4	97	5.3	0.06
Outcome					
Mortality	17	17.7	124	6.8	< 0.01

**Table 3:** Comorbidities among DM and non-DM patients

Comorbidities	Known DM		No known DM		p-value*
	n	%	n	%	
Total	96	100	1816	100	
Myocardial infarct	6	0.6	361	2.0	< 0.01
Heart failure	12	12.5	30	1.7	< 0.01
Peripheral vascular disease	10	10.4	32	1.8	< 0.01
Cerebral vascular disease	15	15.6	70	3.9	< 0.01
Dementia	0	0.0	5	0.3	1
Chronic pulmonary disease	18	18.8	198	10.9	0.02
Connective tissue disease	8	8.3	57	3.1	< 0.01
Ulcer disease	10	10.4	80	4.4	0.01
Mild liver disease	10	10.4	96	5.3	0.05
Moderate/severe renal disease	0	0.0	36	2.0	0.23
Hemiplegia	0	0.0	5	0.3	1
Tumour	11	11.5	78	4.3	< 0.01
Leukaemia	0	0.0	5	0.3	1
Lymphoma	1	1.0	9	0.5	1
Moderate/severe liver disease	4	4.2	10	0.6	< 0.01
Metastatic/solid tumour	0	0.0	6	0.3	1
AIDS	2	2.1	40	2.2	1
Charlson Comorbidity Score n, (%)					
0–1	59	6.2	1561	85.9	< 0.01**
2–3	23	23.9	174	9.6	< 0.01
4–5	8	8.3	34	1.9	< 0.01
= > 6	5	5.2	47	2.6	0.18

*p-value from Chi-square test

**p-value < 0.05 was considered significant, when comparing the percentage of DM patients and no-DM patients stratified by Charlson Comorbidity Score

**Fig. 2:**
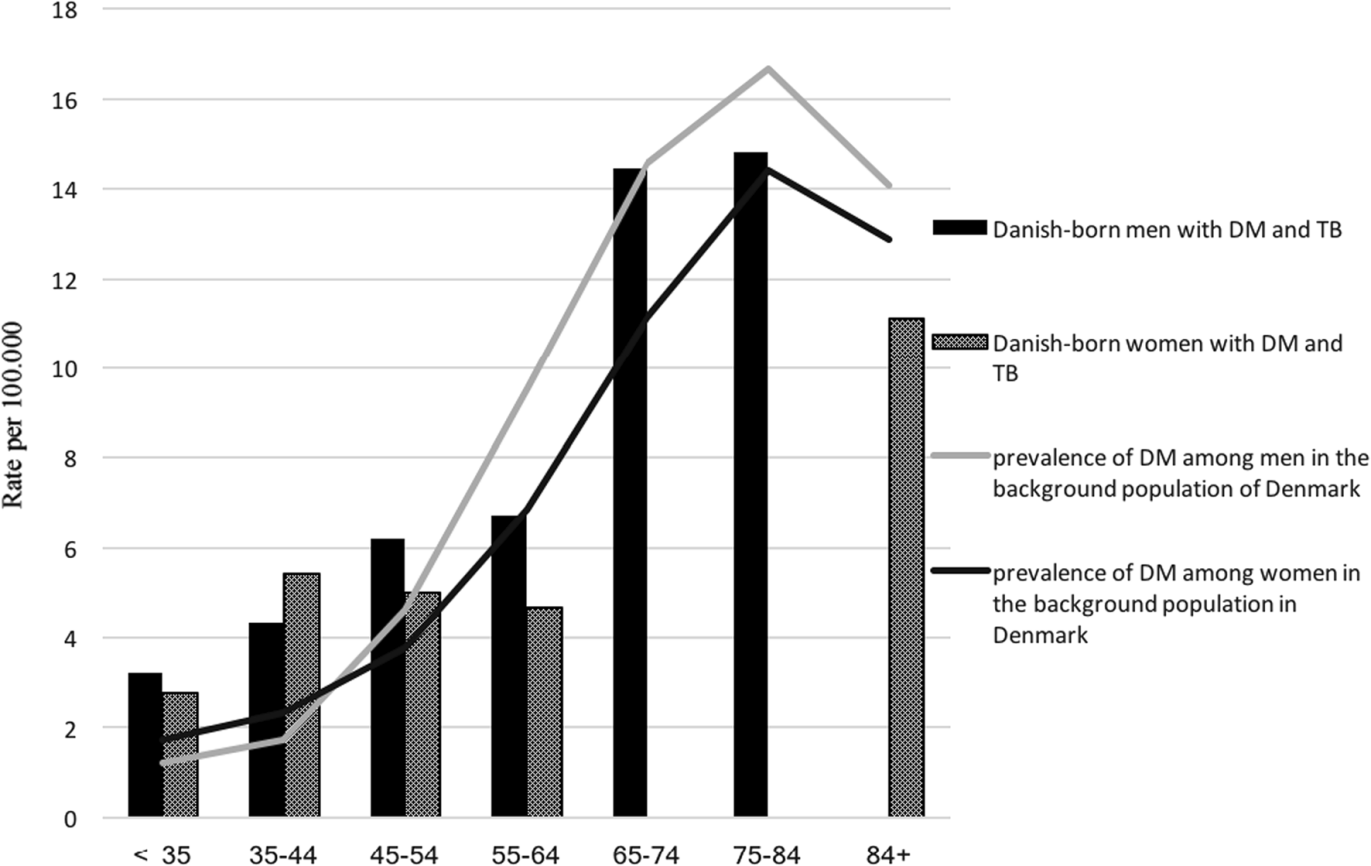
Prevalence (rate per 100.000) of DM among Danish-born and migrant TB patients, compared to the prevalence in the background population of Denmark, stratified by age group and sex

The prevalence of DM among Danish-born TB patients was stratified by sex and age groups and compared with the DM prevalence in the background population in Denmark in 2012^1^ (Fig. 2).

The prevalence of DM among younger TB patients was generally low. Although, young Danish-born TB patients seemed to have a higher prevalence of DM compared to the background population in Denmark (Fig. 2). We found that the prevalence among Danish-born male TB patients aged < 35–54 ranged between 3.2% and 6.2%, compared to 1.7% and 4.6% in the background population. Among Danish-born women < 45 years the DM prevalence ranged from 2.7 to 5.4% compared to 1.7–2.3% in the background population. The two cohorts are not directly comparable, and we did not attempt to analyse statistically significant differences, but the findings could indicate an almost two times increased risk of DM among young TB patients of similar age and sex compared to the background population. Among migrants, we saw a high prevalence of DM among older patients of up to almost 39% among patients aged > 65 years (data not shown). In contrast, Danish-born TB patients ≥ 65 years did not have a higher DM prevalence compared to the Danish background population.

### Risk factors for DM

Risk factor analysis for having DM among TB patients is shown in Table 4. Age above 40 years was statistically significant in both crude (OR 3.5 [2.12; 6.18] p < 0.01) and multivariable analyses (OR 3.49 [2.09; 6.11] p < 0.01) while parameters associated with low socioeconomic status (homelessness, alcohol use, cannabis use, tobacco use) were associated with lower risk of DM (Table 4). Being from another European country than Denmark, from Africa or from Asia was statistically significantly associated with having DM in the multivariable analysis, whereas none of the patients from Greenland had DM and the OR could not be calculated.

**Table 4:** Risk factors for having DM among TB patients

Risk factor	Crude analysis		Multivariable analysis*	
	OR [95%CI]	p-value	OR [95%CI]	p-value
Age				
Age ≤ 40 years	1 (Ref)		1 (Ref)	
Age > 40 years	3.5 [2.12; 6.18]	< 0.01	3.49 [2.09; 6.11]	< 0.01
Sex				
Female	1 (Ref)		1 (Ref)	
Male	1.29 [0.84; 2.02]	0.26	1.21 [0.78; 1.91]	0.4
Socio-economic risk factors				
No alcohol	1 (Ref)		1 (Ref)	
Alcohol	0.41 [0.41; 0.42]	< 0.01	0.25 [0.14; 0.42]	< 0.01
No tobacco	1 (Ref)		1 (Ref)	
Tobacco	0.49 [0.23; 0.57]	< 0.01	0.36 [0.23; 0.57]	< 0.01
No cannabis	1 (Ref)		1 (Ref)	
Cannabis	0.21 [0.07; 0.47]	< 0.01	0.21 [0.07; 0.47]	< 0.01
No narcotics	1 (Ref)		1 (Ref)	
Narcotics	0.83 [0.36; 1.87]	0.65	0.89 [0.36; 1.87]	0.78
Not homeless	1 (Ref)		1 (Ref)	
Homeless	0.36 [0.12; 0.88]	0.05	0.39 [0.12; 0.96]	0.07
No prison time	1 (Ref)		1 (Ref)	
Prison time	< 0.01 [< 0.01; > 149]	0.9	< 0.01 [< 0.01; > 406]	0.98
Country or region of origin				
Denmark	1 (Ref)		1 (Ref)	
Europe	1.68 [0.92; 2.9]	0.07	2.13 [1.13; 3.75]	0.01
Africa	1.42 [0.79; 2.41]	< 0.01	3.3 [1.7; 5.9]	< 0.01
Asia	1.08 [ 0.66; 1.71]	0.75	1.8 [1.09; 2.99]	0.02
Greenland**	Not applicable	–	Not applicable	–
South America	Not applicable	–	Not applicable	–
North America	Not applicable	*–*	Not applicable	–

*Adjusted for age, sex, not including the measured risk factor

**Due to the fact that there were no patients from Greenland with DM the OR = 0.0, and p-value could not be assessed. The same was the case for North and South America

## Discussion

We found an overall prevalence of DM of 5.0% among TB patients in Denmark. The prevalence among Danish-born TB patients was 6.1%. Young Danish-born TB patients seemed to have a higher prevalence of DM compared to an age and sex matched Danish background population and a high proportion, up to 39%, of older migrant TB patients had DM. Age over 40 years and being from Africa, Europe outside Denmark, or Asia were risk factors for having DM, whereas being from Greenland was not. Factors related to low socio-economic status were associated with not having DM.

The DM prevalence ranged from 0% among patients from Greenland, 6.1% among patients from Denmark to 7.7% among patients from Europe, outside Denmark. The prevalence of 6.1% among Danish-born was slightly higher than the estimated prevalence of 4.9% among the Danish background population [18]. The risk of TB among patients with DM in Denmark has been addressed in a register-based population study [19] showing a high rate ratio (RR) of TB (RR: 1.9, 95% CI [1.7–2.1]) among DM patients compared to non-DM patients. In contrast to our study, they found a low RR (0.5) for having TB among African DM patients when stratifying for ethnicities. The apparent differences could be explained by differences of age, study design and study period.

The DM prevalence among older migrants was as expected higher compared to the Danish patients. This is in line with studies from Africa, Asia, and countries such as Mexico, reporting prevalences of DM among TB patients ranging from 5% to up to 50% [20–23]. Interestingly, these studies found that the DM prevalence among TB patients was not only high, but also higher than in the background population. The high prevalence not only reflects the high burden of DM in the areas, it also underlines the possible interaction between the two diseases where DM and poor glycaemic control compromise innate and adaptive immunity and enhance susceptibility to TB [23, 24].

The finding that the prevalence of DM among migrant TB patients was highest in the old age groups could be related to the fact that DM is associated both with age and the fact that many patients migrated from DM high-endemic regions and therefore had a higher risk of DM compared to the Danish patients. Also, older TB patients also had more comorbidities and therefore also had more contact with the health-care system allowing them to be diagnosed with DM [26].

In contrast the lower than expected prevalence in the young migrant TB patients could be due to differences in health seeking behaviour explained by cultural differences, language barriers or social problems such as homelessness and substance abuse or the fact that they were newly immigrants to Denmark. Patients from Greenland were included in the migration-group, in the assessment of age, sex and DM in Fig. 2.

In our study, younger Danish-born women (35–44 years of age) and men (35–54 years of age) had almost two times higher prevalence of DM compared to an age matched background population. This was an unexpected finding, and even though the numbers are small and the two cohorts are not directly comparable, one should be aware of the possible interactions in younger individuals, and patients with undiagnosed DM and pre-DM may be at risk of complications to DM if undiagnosed and untreated. Together these findings emphasize the need for DM screening of TB patients even in a low incidence country as Denmark. Danish guidelines for TB [25, 27] focus on this matter by recommending screening of TB patients for DM and suggest treatment of latent TB infection in individuals from high TB endemic regions with compromised immunity including DM.

In Denmark, TB is frequently seen among individuals from Greenland where the incidence of TB is high [28]. We found no patients with known DM among TB patients from Greenland. This is surprising, since other studies show a high (and increasing) prevalence of DM in Greenland. One study [29] found a DM prevalence of between 15 and 18% among the Inuit population in Greenland, when using an oral glucose test, and between 6.2 and 6.8%, when using HbA1c. Additionally, a relative risk for TB among DM patients in Greenland of 11.7 has been reported, and DM in TB patients from Greenland would be expected to be common [30]. One reason for the low prevalence in patients from Greenland could be due to lack of knowledge of their DM status; it has previously been estimated that 70% of Greenlanders with DM were unaware of their DM status [31]. This estimate, however, is almost 20 years old and awareness of DM both in the Greenlandic public and health care system has increased in recent years [32].

The patients from Greenland in this study had a high prevalence of homelessness, tobacco use and alcohol use. This was also true for patients from Denmark. Homelessness, tobacco use and alcohol use is associated with low socioeconomic status. These patients often experience social barriers and therefore have altered health seeking behaviour [33–36], which could be a reason why these patients were not screened for DM. Danish studies among socially marginalised individuals show a high use of both primary and secondary health care services, but only in relation to critical illness [34, 35, 37].

Thus, the low prevalence of known DM among TB patients from Greenland could be explained by low socio-economic status and result in an underestimation of the DM prevalence in Greenlanders. Since we suspect that the patients from Greenland were underdiagnosed with DM, we believe that the prevalence of DM in the migration-group was biased towards a lower prevalence than the estimated prevalence.

As a consequence of this study and the increased awareness of the potential severe outcome of TB in DM patients, we are now routinely screening all TB patients for DM with HbA1c and blood glucose at TB diagnosis in line with screening for HIV and Hepatitis. Preliminary findings showed that 1.8% were newly diagnosed with DM and that 27.3% had pre-DM with blood glucose of > 39 mmol/mol [38]. These patients are referred for treatment of DM and are informed about risk reduction and advised a control by their general practitioner.

### Strengths and limitations

The major strength of this study was that we were able to identify all patients diagnosed with TB in Denmark between 2009 and 2014 due to the close TB monitoring performed by SSI and the Department of Infectious Epidemiology and Prevention.

In Denmark, TB treatment is free of charge and all patients are treated regardless of their residence status. We can thus assume that no patients opted out of treatment or were not treated due to financial reasons.

The main limitation of this study is the retrospective study design, with information gathered only from patient records and therefore we were only able to include information on DM registered in a Danish hospital medical record at the time of TB diagnosis. Thus, we have no information on DM diagnosis made after the start of treatment or in other settings such as by a primary physician and our estimations of DM prevalence are most likely underestimated. Also, we did not have access to information on family status, lifestyle or how long patients had had their DM diagnosis prior to TB diagnosis, which could be a confounding issue. Since DM diagnosis was not uniformly registered, this may have resulted in the fact that some individuals may have been misclassified resulting in underestimation of the DM prevalence. We were not able to analyse the prevalence of pre-DM or dysglycemia since we did not have HbA1c levels or blood glucose on all patients. To explore the true magnitude of the DM problem among TB patients in Denmark, there is a call for prospective studies including a follow up period, where different aspects of DM, pre-diabetes and dysglycemia can be assessed.

## Conclusion

To our knowledge this study is the first study to address the prevalence of DM among TB patients in Denmark. The prevalence of DM was slightly higher in the Danish background population and young Danish-born TB patients had an increased risk of DM, calling for follow up studies. Age over 40 years and being from Africa, Europe outside Denmark, and Asia were risk factors for having DM, whereas no patients from Greenland had known DM. Factors associated with low socio-economic status were associated with not having DM.

We find a need for prospective studies addressing the actual risk of DM and pre-DM among patients with TB in Denmark in order to diagnose patients at risk of DM in due time.

## Data Availability

The dataset used and/or analysed during the current study are available from the corresponding author on reasonable request.

## Abbreviations

TB: Tuberculosis
DM: Diabetes mellitus
SSI: Statens Serum Institut
LPR: Landspatientregister
CPR: Central person register

## Footnotes

1 Flachs EM, Eriksen L, Koch MB, Ryd JT, Dibba E, Ettrup L, Juel K. Statens Institut for Folkesundhed, Syddansk Universitet. Sygdomsbyrden i Danmark – sygdomme. København: Sundhedsstyrelsen; 2015.
